# A single recall vaccination lapse in sows triggers PRRSV resurgence and boosts viral genetic diversity

**DOI:** 10.1186/s40813-025-00433-w

**Published:** 2025-05-08

**Authors:** H. Clilverd, G. E. Martín-Valls, Y. Li, I. Domingo-Carreño, M. Martín, M. Cortey, E. Mateu

**Affiliations:** Departament de Sanitat i Anatomia Animals, Universitat Autònoma de Barcelona, Travessera dels Turons s/n, 08193 Cerdanyola del Vallès, Bellaterra, Spain

**Keywords:** Porcine reproductive and respiratory syndrome virus (PRRSV), Vaccination, Genetic diversity, Evolution

## Abstract

**Background:**

Porcine reproductive and respiratory syndrome virus (PRRSV) persists on certain farms despite vaccination and control efforts, with genetic diversity suspected as a contributing factor. This study examined the evolution and persistence dynamics of PRRSV-1 on a farrow-to-fattening farm with 1,700 sows vaccinated quarterly, focusing on a summer vaccination lapse.

**Results:**

Over eight months, three farrowing batches were monitored from birth to nine weeks of age using virological (RT-qPCR, whole-genome, and ORF5 sequencing) and serological (ELISA and neutralizing antibody) analyses. An incident related to elevated temperatures during the summer involving unproper vaccine handling occurred during the last blanket vaccination, before sampling the third batch. Viral circulation was primarily confined to the nurseries, with a notable surge of incidence and mortality in this last batch, linked to lower maternal antibody levels likely due to vaccination failure. Phylogenetic analyses showed the persistence of the same viral strain throughout the study, with increased genetic diversity in Batch 3 driven by selection and recombination. Ultimately, reestablishing the vaccination program led to a PRRSV-positive-stable with vaccination status.

**Conclusions:**

Overall, a single vaccination lapse caused increased PRRSV-1 incidence and genetic diversity in weaners, linked to declining maternal antibody levels, underscoring the importance of strict vaccination adherence.

**Supplementary Information:**

The online version contains supplementary material available at 10.1186/s40813-025-00433-w.

## Background

Porcine reproductive and respiratory syndrome virus (PRRSV) infection causes severe economic losses in affected farms [1–3]. Following the introduction of the virus into a breeding herd, the infection usually establishes an endemic cycle [4], where susceptible sows give birth to viraemic piglets that perpetuate the infection in nurseries. A key strategy for controlling PRRSV is implementing a robust vaccination program in sows to limit vertical transmission, thereby stabilizing the herd. Once the stabilisation is achieved, eliminating the infection from nurseries may necessitate partial depopulation [5]. Despite these measures, some herds experience new outbreaks or rebounds in PRRSV incidence, often involving the resurgence of the same viral strain. Various factors may contribute to these re-emergences of the resident PRRSV strain, being the presence of non-immune breeders one of the main circumstances. This study investigates the consequences of a single lapse in recall vaccination compliance within a breeding herd, focusing on its role in PRRSV resurgence and amplification of viral genetic diversity.

## Methods

### Case farm and follow-up chronology

A 1,700-sow farrow-to-fattening farm was being monitored in the frame of a viral evolution study. The farm operated on a one-week farrowing batch schedule, with approximately 80 sows per batch. Replacement gilts were purchased from a PRRSV-negative farm at 5.5 months of age, and subsequently introduced into the breeding herd following a six-week quarantine. The PRRSV vaccination plan included two doses of a modified live vaccine (PYRSVAC-183^®^, SYVA Laboratories) during the acclimatisation period, along with quarterly recall vaccinations to the breeders with the same vaccine.

The ongoing viral evolution study aimed to examine the evolution of the circulating PRRSV from birth to the end of the nursery phase in endemic farms. To accomplish this, animals from three farrowing batches (designated as batches 1–3) were monitored over an eight-month period. Sampled sows corresponded to randomly selected farrowing rooms (20–24 sows/room). Within each batch piglets were selected ra For Batch 1, sows were vaccinated 4 weeks before farrowing; for Batch 2, they were vaccinated 8 weeks before farrowing, while the recall PRRSV vaccination of Batch 3 sows was scheduled six weeks before the expected farrowing date; namely, before day 70 of gestation. This vaccination took place in late July, coinciding with maximum temperatures exceeding 35 °C. Ventilation in the gestation barns was operated manually. During the vaccination process, the reconstituted vaccine was left at room temperature for several hours. Although a precise record of the internal barn temperatures is unavailable, the room temperature exceeded the comfort range for the sows and the recommended conditions for conserving the reconstituted vaccine.

### Sampling and PRRSV detection

In the three examined farrowing batches, a total of 535 piglets from 151 sows were followed from birth to nine weeks of age. The second batch was examined 4 weeks after the first and the third was examined 17 weeks after the second, namely 21 weeks after the first. Samples (at least 4 piglets per examined litter) were collected at birth (umbilical cords, UC), with subsequent blood collection from the piglets at three, six, and nine weeks of age. UC were processed following a previously described protocol [6] and blood samples underwent centrifugation at 300 g for five minutes before storage at -80 °C. All blood samples were analysed individually. For UC, pools of two samples were initially analysed and if the pool tested positive individual samples were re-tested.

Viral RNA extraction was conducted using MagMax Core Nucleic Acid Purification 264 Kit (Applied Biosystems, ThermoFisher Scientific, United States), following the manufacturer’s instructions. The PRRSV status was determined using a commercial RT-qPCR kit (VetMAX™ PRRSV EU & NA 2.0 Kit, ThermoFisher Scientific), including an internal positive control in each sample. Samples with C_t_ values < 37 were considered positive.

Vertical transmission was determined by identifying cases where at least one positive UC per litter was detected by RT-qPCR. Cumulative incidence rates were calculated by dividing the number of new cases by the number of susceptible animals through each observation period. Calculations excluded animals for which data was incomplete or unavailable.

### Serological analyses

Anti-PRRSV antibodies were determined in all serum samples of three-week-old piglets through a commercial ELISA kit (IDEXX PRRS X3 Ab Test, IDEXX, United States). Additionally, the level of neutralizing antibodies was determined in 50 three-week-old piglets per batch matched by parity of the sow and randomly selected from those testing negative by RT-qPCR up to that age. The VNT was conducted according to Yoon et al. [7] with minor modifications using either the vaccine strain used on the farm, or the strain circulating in that farm and adapted to grow in MARC-145 cells. To assess whether changes in the anti-PRRSV antibody titres reflected a general decrease in maternal immunity, sera of the offspring of the fourteen sows present in both batches 1 and 3 were analysed for anti-pseudorabies total antibodies by ELISA (Ingezim^®^ ADV Total, Gold Standard Diagnostics, Madrid), as the sows were vaccinated with a pseudorabies gE-deleted MLV every four months, but not coincidently with PRRSV vaccination.

### Sequencing and phylogenetic analyses

Samples with C_t_ values < 32 (all in Batch 1 and 2 and 50% in Batch 3) were ORF5 Sanger sequenced, following a previously described protocol with minor modifications [8]. Viral isolation in porcine alveolar macrophages (PAM) was conducted for at least 20% of the sequenced samples, and the resulting cell-culture supernatants underwent whole-genome sequencing (WGS) using Illumina MiSeq RNAseq, in accordance with a previously detailed protocol [9].

The consensus sequences obtained from both whole genome (*n* = 55) and the ORF5 (*n* = 213) sequencing were submitted to GenBank with the Accession Numbers PP261834 to PP261888, and PP261642 to PP261833, respectively.

A subset of sequence analyses was performed including: the construction of phylogenetic trees through Bayesian inference using Mr. Bayes [10] (available at https://ngphylogeny.fr); the determination of nucleotide identities within and between clades with p-distance using MEGA XI [11]; the prediction of N-glycosylations in the viral glycoproteins using Net-N-Glyc 1.0 [12] (available at: https://services.healthtech.dtu.dk/services/NetNGlyc-1.0/.); and the evaluation of recombination patterns using GARD algorithm method [13]. In addition, the amino acid composition of the predicted sequences was compared between the identified clades.

### Statistical analyses

PRRSV incidences were compared using the χ^2^ test (Fisher’s exact test). Comparison of S/P ratios and levels of neutralizing antibodies were performed using Kruskal-Wallis test and t-test. All statistical analyses were conducted using GraphPad Prism v10, with significance set at *p* < 0.05.

## Results

At the beginning of the study, in Batch 1, viral circulation was primarily restricted to the nurseries, with sporadic occurrences of vertical transmission (Table 1). In the following batch, Batch 2, the incidences at weaning and in nurseries decreased. A significant rise in PRRSV circulation was observed in the third batch, mainly affecting six- and nine-week-old pigs (from 6.1 to 98.8% incidence at six weeks of age in Batch 2 and 3), while vertical transmission remained unaffected.

**Table 1:** Data summary for each batch. The table shows the data of the monitored animals, vertical transmission frequency, and cumulative incidences at three, six, and nine weeks of age for each followed batch

Batch	Nº litters	Range of sows’ parities	Pigs followed (1–9 woa)	Litters with PRRS-positive piglets at birth	Incidence at 3 woa	Incidence at 6 woa	Incidence at 9 woa
**1**	44	1–9	170	1 (2.3%) ^a^ (CI_95%_: 0.1–13.5%)	6.5% ^a^	19.9% ^b^	60.8% ^b^
**2**	42	1–9	173	3 (7.1%) ^a^ (CI_95%_: 1.9–20.6%)	4.1% ^a^	6.1% ^c^	20.9% ^c^
**3**	65	1–8	192	3 (4.6%) ^a^ (CI_95%_: 1.2–13.6%)	7.3% ^a^	98.8% ^a^	100.0% ^a^
**Totals/ Average**	**151**	**1–9**	**535**	**4.6%**	**N.A.**	**N.A.**	**N.A.**

woa = weeks of age. N.A. non-applicable. Values with a different superscript letter indicate significant differences (*p* < 0.05)

**Fig. 1:**
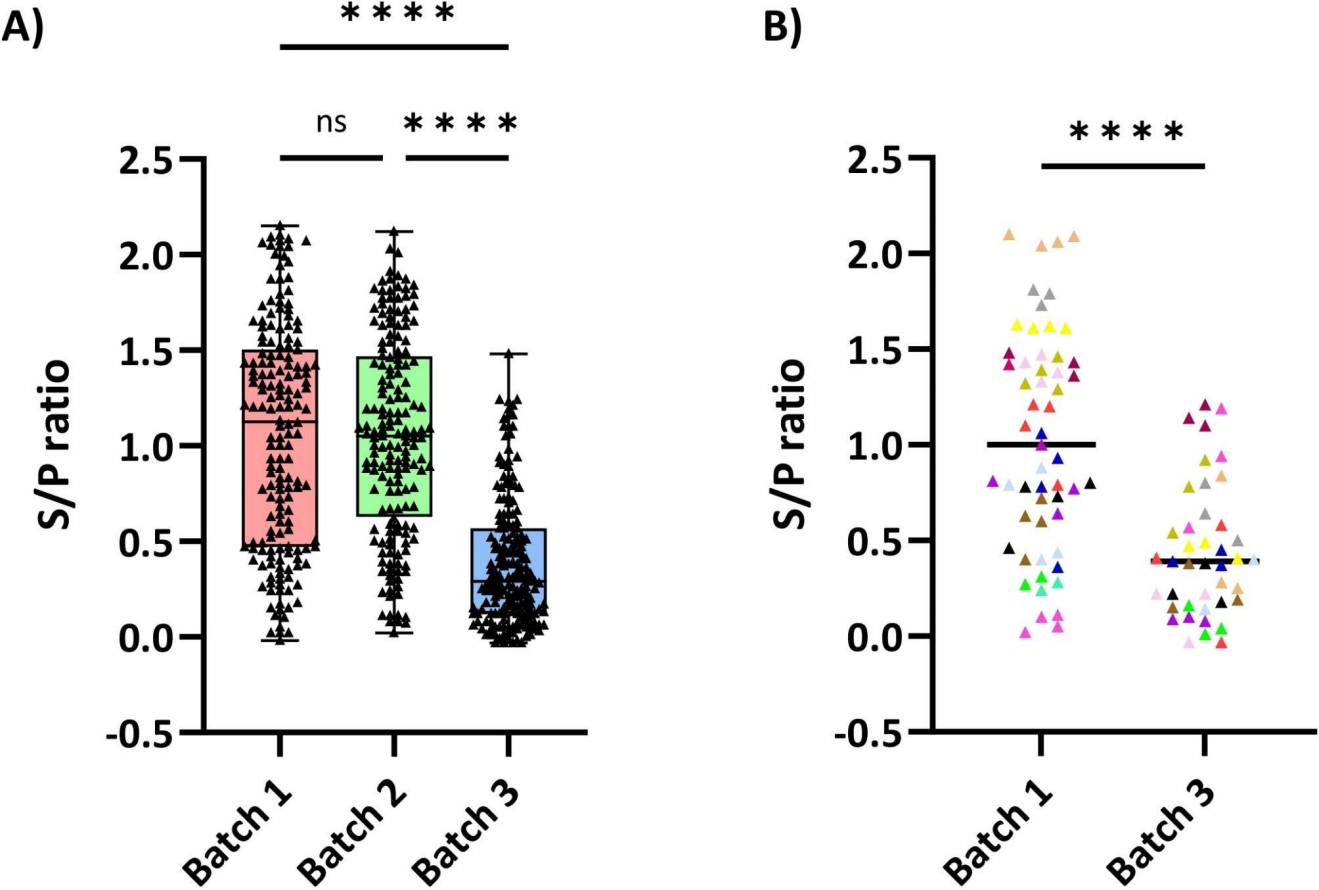
PRRSV-antibody levels of three-week-old piglets as determined by ELISA (S/P ratios). Each triangle represents an individual. S/P ratios ≥ 0.4 are considered positive. **(A)** Distribution of S/P ratios for all examined animals per batch. **(B)** Distribution of S/P ratios for the offspring of the fourteen sows present in batches 1 and 3. Offspring from the same sow are depicted using the same colour. ns = non-significant; *p < 0.05; **p < 0.01; ***p < 0.001; ****p < 0.0001

In Batch 3, the proportion of seropositive piglets at three weeks of age dropped significantly compared to earlier batches (83% and 88% in batches 1 and 2 vs. 37% in Batch 3, *p* < 0.05) (Fig. 1A), regardless the sow’s parity (data not shown). Interestingly, a group of fourteen sows were sampled in both batches 1 and 3. The average S/P ratios of their piglets at three weeks of age were significantly different between batches (1.03 ± 0.08 vs. 0.44 ± 0.35, respectively; *p* < 0.0001), indicating a decrease in the levels of PRRSV antibodies in Batch 3 compared to Batch 1. Figure 1B shows the individual S/P ratios of the offspring of the 14 sows sampled in both batches. When the same sera were examined for anti-pseudorabies antibodies no differences were found between the S/P values of their piglets [see Additional file 1]. Additionally, the average S/P ratios of the fourteen litters were not significantly different between batches (1.41 ± 0.19 vs. 1.42 ± 0.24, respectively; *p* = 0.88).

**Fig. 2:**
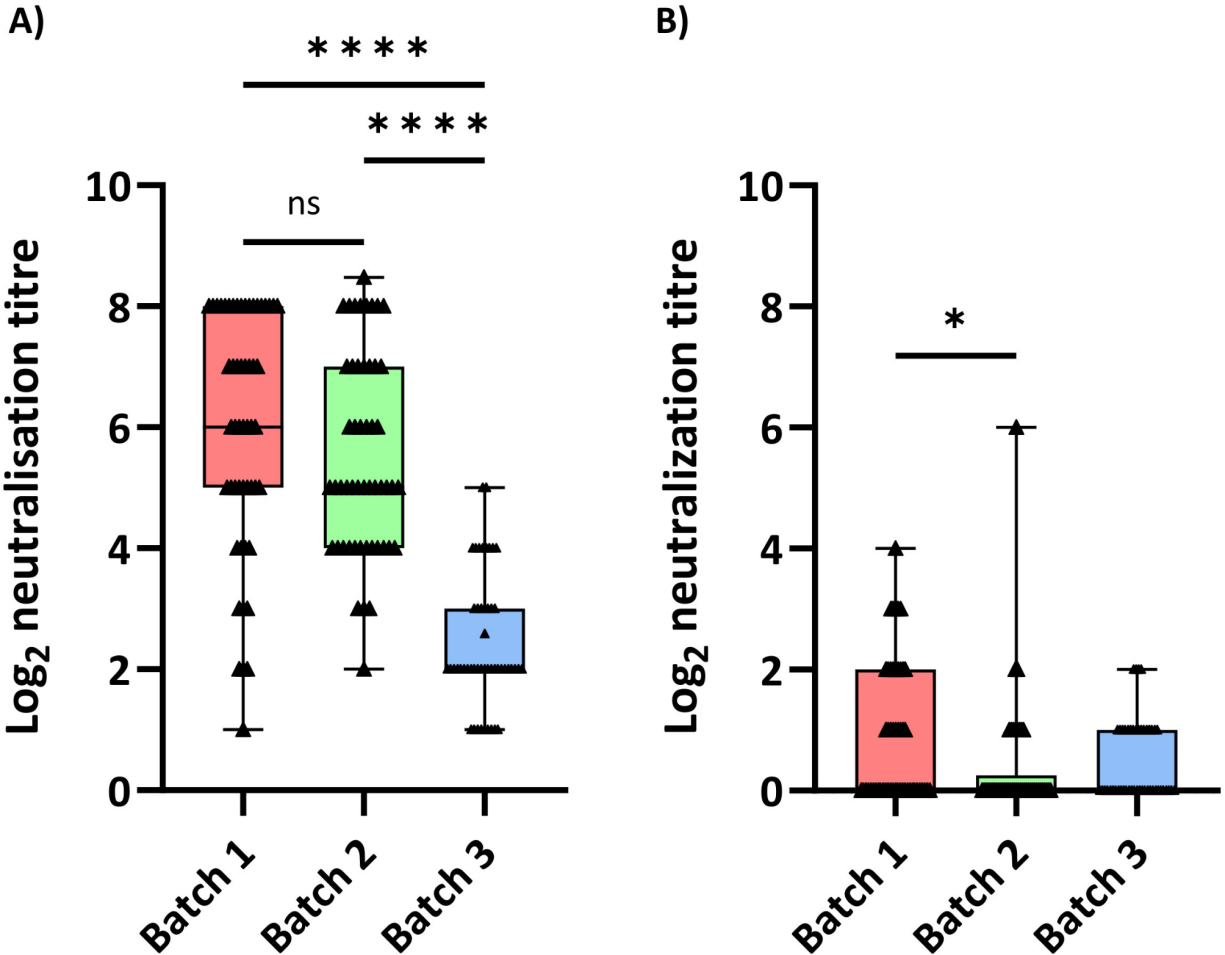
PRRSV-neutralizing-antibody titres (log_2_) of three-week old piglets as determined by virus neutralization test. **(A)** Results of the VNT analysis of piglets in batches 1, 2, and 3 using the vaccine strain. **(B)** Results of the same animals when using the PRRSV strain circulating in the farm adapted to grow in MARC-145 cells. Each triangle represents an individual. ns = non-significant; *p < 0.05; **p < 0.01; ***p < 0.001; ****p < 0.0001

Neutralizing antibodies were evaluated in 50 randomly selected, three-week-old piglets with no prior infection up to that age. The selected animals were distributed proportionally by sow parity grouped as young (parities 1–2, mature, parities 3–6 and old, parity ≥ 7). The results (Fig. 2) showed that median neutralization titres against the vaccine virus ranged from 5 to 6 log_2_ in batches 1 and 2, whereas Batch 3 exhibited a significantly lower median titre of 2.0 log_2_ (*p* < 0.05). This decrease could be attributed to vaccination failure during the summer months. For the field PRRSV strain, VNT results indicated very little induction of neutralizing antibodies, with more than 50% of the animals testing negative in the PRRSV VNT across all three batches.

**Fig. 3:**
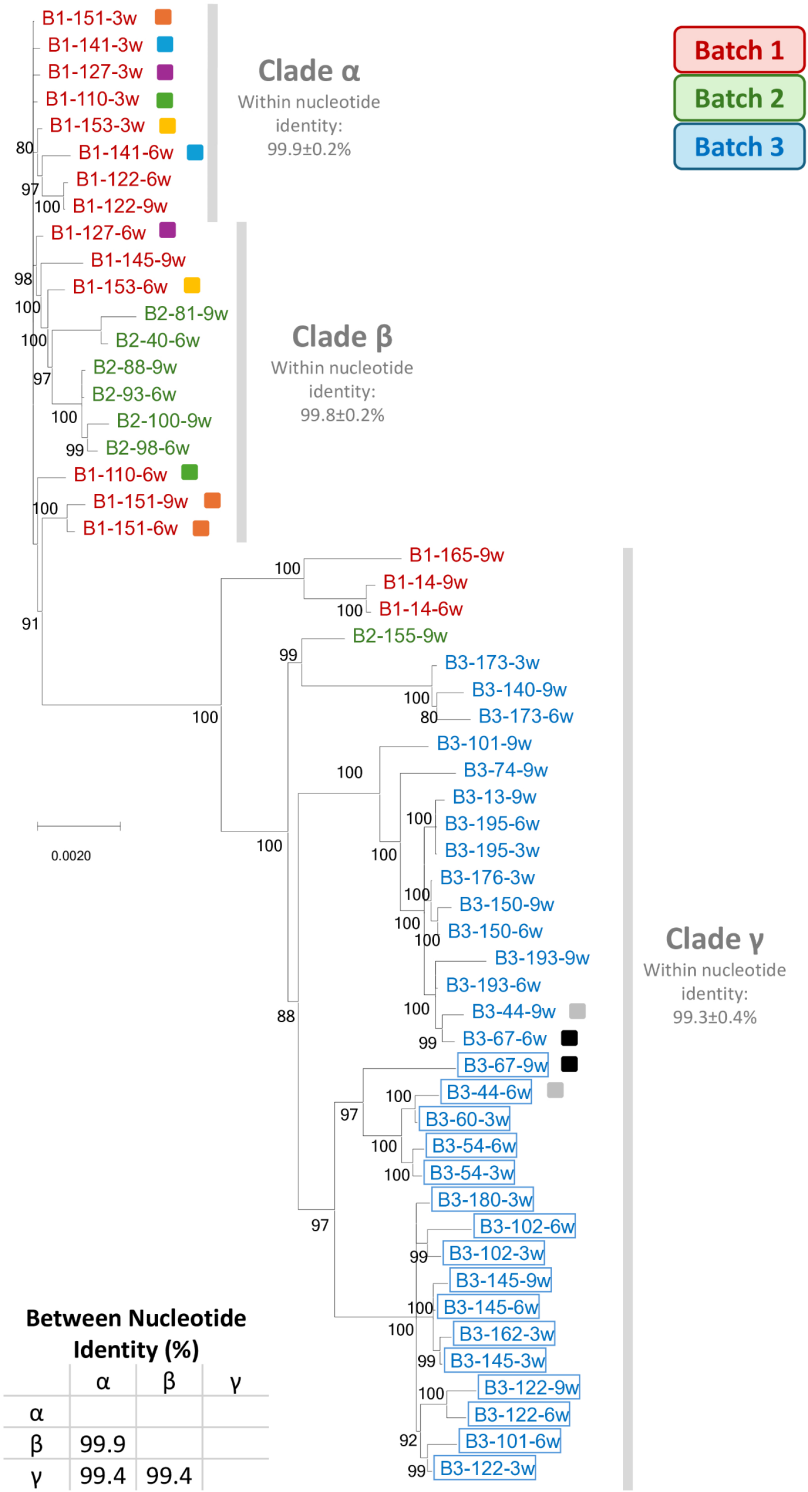
Bayesian analysis of the whole genome sequences obtained in this study. Posterior probabilities higher than 70% are shown. The colour-coded representation designates sequences retrieved from animals of Batches 1, 2, and 3 in red, green, and blue, respectively. Individuals whose isolates from different ages clustered into a different clade are indicated with the same-coloured square. Blue-coloured boxes indicate isolates harbouring a truncated GP3

Overall, the number of full genome sequences produced was 55 (17 for Batch 1, 7 for Batch 2 and 31 for Batch 3) and 247 ORF5 sequences (66 for Batch 1, 29 for Batch 2 and 152 for Batch 3). Phylogenetic analyses of the complete genome (Fig. 3) and ORF5 [see Additional file 2] consistently identified the same viral strain throughout the study period, identifying distinct circulating clades. The overall nucleotide identity for the whole viral genomes retrieved from all three batches was 99.2% ± 0.04%, with none matching the vaccine strain used on the farm (86.2% nucleotide identity). In Batch 1, at least three circulating clades -designated α, β, and γ- were distinguished, though clade α was not detected in subsequent batches. For Batch 2, clade β dominated, infecting all animals except one, which was infected by γ. In Batch 3, the γ clade prevailed, being the only one detected. Interestingly, Genetic diversity within Batch 3 - (0.54% ± 0.04%) was higher compared to Batch 1 (0.37% ± 0.01%) and 2 (0.39% ± 0.03%), coinciding with the sharp increase in PRRSV incidence at six weeks of age. Notably, in Batch 3, mortality in the nurseries rose to 15% from the previous average of less than 2%.

Clade γ differed from the other clades at 83 amino acid positions throughout the genome, of which 36 were fixed amino acid substitutions. These 36 fixed amino acid substitutions specific for this clade accumulated mostly in nsp2 (11), nsp10 (4), GP3 (4), and M protein (4), representing a 1.1%, 0.9%, 2.3%, and 1.5% of each protein, respectively. The remaining 47 variable amino acids sites identified in clade γ appeared mostly, but not exclusively, in Batch 3 isolates (41/47) and accumulated mainly in nsp2 (20), nsp5 (4), nsp1b (4), and nsp3 (3) (2.0%, 2.4%, 1.6%, and 1.0% of each protein, respectively). Although variability was observed in GP5, none of the non-synonymous mutations was present in all γ clade isolates. Clade γ had an additional potential N-glycosylation site in GP3 at position 27 compared to the amino acid sequence of Lelystad virus (LV, Genbank accession number NC_043487).

It is worth noting that sixteen isolates in clade γ harboured a truncated GP3 protein resulting from a stop codon (position 241 corresponding to LV). Truncation was confirmed by ORF3 Sanger sequencing. Sequences with this truncation clustered together within the γ clade. Moreover, isolates from all batches harboured an amino acid deletion in GP3 at position 246 and in GP4 at position 66, corresponding to LV, both located in described neutralizing epitopes [14, 15].

Moreover, the analyses revealed that, in seven animals, isolates obtained at different ages from the same individual grouped into distinct clades. Recombination analyses suggested that the differences in clustering observed in four of these cases (sequences B1-141, B1-151; B1-153, and B3-67 in Fig. 3) might potentially result from genetic recombination between different clades, while in one case (B3-44), it could be attributed to a reinfection from a different clade. The distinct classification for the remaining two individuals (B1-110; B1-127) cannot be definitively determined [see Additional file 3].

Noteworthy, the reestablishment of the vaccination program eventually led the farm to achieve a PRRSV-positive-stable with vaccination status one year after Batch 3 (data not shown).

## Discussion

A proper handling of vaccines and a compliant vaccination procedure is essential to ensure the success of vaccination. In the case of PRRSV, the virus easily inactivates at high temperatures; as shown previously, half-life at 37 °C does not exceed 3 h [16]. In our case, before the vaccination issue arose, the PRRS control program in the farm was limiting the spread of the infection at early ages. Despite vertical transmission was detected in 2–7% of litters, horizontal transmission in nurseries remained limited and the reproductive performance was acceptable. This limitation in the transmission was probably attributable to the maternally-derived immunity, together with proper management practices of nurseries.

The fortuitous coincidence of the vaccination issue with one of the batches under observation provided the opportunity to examine on field the impact of unproper recall vaccination on the incidence and the genetic diversity of PRRSV. As evidenced by the serological results, just one vaccination incident affecting sows in Batch 3 resulted in a dramatic drop of maternally-derived antibodies in piglets. This decline occurred for all sows, regardless of parity, indicating that the reduction in antibody titres was not mitigated by the number of vaccine doses received in the past. Furthermore, pseudorabies ELISA results demonstrated that this drop was specific for PRRSV, indicating that vaccination failure was the most likely cause, rather than an external factor impacting the overall immune performance of the sows, such as the thermal stress experienced by the sows arising from the elevated temperatures during that period. The decline in the antibody titres probably facilitated an earlier and wider spread of the virus in nurseries, with almost all piglets becoming infected between three and six weeks of age. Since no other changes were recorded in the management of the farm, the unproper vaccine handling stands out as the most likely explanation.

In a farm like the one examined in this case, most of the circulating virus is produced in the nurseries, where hundreds of animals can become infected in a short period of time. Thus, infected nurseries can be sources of infection for the sows present on the same farm, either by airborne transmission or by contact with fomites, particularly if biosecurity measures are not extremely strict. The VNT results revealed that the progressive reduction in the circulation of the wild-type virus following Batch 1 led to a rapid decline in the neutralizing antibodies specific for the resident PRRSV strain in sows, suggesting that in the absence of viral circulation, or in a scenario where it was very limited, there was a rapid decline of the humoral response, consistently with a short-lived memory response for neutralizing antibody-producing clones.

Additionally, this increased transmission was coupled with an increase of genetic diversity of the virus. This phenomenon aligns with the larger number of infected animals at early ages, with almost all pigs in Batch 3 being infected at six weeks of age, contributing to the subsequent expansion of the viral cloud. This fact underscores the importance of a strict adherence to vaccination schedules in endemic herds. While it can be argued that infected animals will eventually reach the nurseries, and sooner or later the virus will spread, field evidence suggests that the earlier the age of infection, the greater the impact of PRRSV on the farm. In our case, this earlier circulation resulted in a sharp increase of the mortality in the nurseries that rose up to 15% in the third batch from less than 2% in batches 1 and 2.

Furthermore, despite the initial circulation of at least three viral clades (α, β, and γ) in this herd, only two clades circulated in the second batch (β and γ), of which only one endured into the third batch, where it underwent further diversification (γ). This suggests the influence of a selection as the evolutionary force that favoured a specific viral clade while extinguishing others, both from Batch 1 to 2 and Batch 2 to 3. It is difficult to guess what would have occurred without the vaccination issue. Although γ-clade isolates could have some better fitness for transmission, it is more likely that the decrease of the immunity was responsible for the increased transmission. Additionally, upon the reintroduction of the vaccination program, the farm achieved a PRRSV-positive stable status, reflecting a significant reduction in the circulation of the newly dominant clade, thereby further reinforcing that idea. Hence, not all variants possess equal capabilities for long-term persistence on farms, and despite their predominance at a given moment, they may vanish when conditions change. Investigating the reasons behind the persistence or disappearance of specific variants could provide valuable epidemiological insights. In our case, passive immunity received by piglets was probably one of the key drivers of the viral evolution.

One notable observation is the identification of a subclade of the γ clade harbouring a truncated GP3 on the 3’ end. The predicted amino acid sequence in this case would be 241 amino acid long. GP3, in conjunction with GP2 and GP4, forms a heterotrimer that interacts with CD163 [17, 18], the essential viral receptor in porcine alveolar macrophages [19]. This finding suggests that the deleted segment would not be essential for such interaction and agrees with findings from previous reports [20–23].

Moreover, among the seven instances where isolates from different timepoints in the same individual formed separate clusters, four were likely the result of recombination. This underscores a notable frequency of interclade recombination events. The occurrence of recombination appears to be more widespread than previously thought, possibly owing to the limitations of previous sequencing methodologies. These limitations both involved only identifying those recombination events within the sequenced segments of the genome and only detecting those recombination variants that became predominant. WGS with current methodologies has brought to light a higher frequency of recombination events that were previously overlooked.

Interestingly, a considerable proportion of sows lack neutralizing antibodies against the circulating strain. In other papers [24–26] it was shown that in vaccinated endemically infected herds is possible to find a proportion of sows not having detectable neutralizing antibodies. This can be the result of some sows having not been in contact with the field virus, a poor induction of this type of antibodies raised against the circulating strain and a matter of idiosyncrasy, as shown by Fiers et al. [27].

## Conclusions

These findings emphasize the critical importance of strictly adhering to vaccination schedules and implementing robust vaccination procedures to exert an effective impact on PRRSV viral circulation. Within this herd, the drop in the humoral immunity, the enhanced transmission, and recombination emerged as substantial driving forces contributing to the genetic diversity of PRRSV.

## Electronic supplementary material

Below is the link to the electronic supplementary material.

Additional file 1. Pseudorabies-antibody levels of three-week-old piglets as determined by ELISA (S/P ratios). Distribution of S/P ratios for the offspring of the fourteen sows present in batches 1 and 3. Each triangle represents an individual. S/P ratios ≥ 0.4 are considered positive. ns = non-significant.

Additional file 2. Bayesian analysis of the ORF5 sequences obtained in this study. Posterior probabilities higher than 70% are shown. The colour-coded representation designates animals from Batches 1, 2, and 3 in red, green, and blue, respectively. Additionally, the identified clades from the complete genome analyses have been marked.

Additional file 3. Recombination analysis of the whole genome sequences obtained in this study. Phylogenetic trees were constructed based on each recombinant fragment, incorporating the whole genome sequences of Batch 1 (in red), 2 (in green), and 3 (in blue). Individuals whose isolates from different ages clustered into a different clade are indicated with the same-coloured square.

## Data Availability

The datasets of sequences are available GenBank and accession numbers (PP261834 to PP261888, and PP261642 to PP261833) can be found in the main text.

## Abbreviations

ADV: Aujeszky Disease Virus
CI: Confidence Interval
C_t_: Cycle threshold
ELISA: Enzyme-linked immunosorbent assay
GP: Glycoprotein
LV: Lelystad Virus
MLV: Modified live vaccine
N.A.: Non-applicable
ns: non-significant
nsp: non-structural protein
ORF: Open-reading frame
PAM: Porcine Alveolar Macrophage
PRRS: Porcine Reproductive and Respiratory Syndrome
PRRSV: Porcine Reproductive and Respiratory Syndrome Virus
RNA: Ribonucleic Acid
RT-qPCR: Retro Transcriptase quantitative Polymerase Chain Reaction
UC: Umbilical Cord
VNT: Virus Neutralization Test
WGS: Whole Genome Sequencing
woa: weeks of age
