# Supplementary material for: A single recall vaccination lapse in sows triggers PRRSV resurgence and boosts viral genetic diversity
Source: Porcine Health Manag. 2025 May 8;11:26. doi: 10.1186/s40813-025-00433-w (PMC12063453; doi:10.1186/s40813-025-00433-w)

Additional File 1. Pseudorabies-antibody levels of three-week-old piglets as determined by ELISA (S/P ratios). Distribution of S/P ratios for the offspring of the fourteen sows present in batches 1 and 3. Each triangle represents an individual. S/P ratios ≥0.4 are considered positive. ns= non-significant.

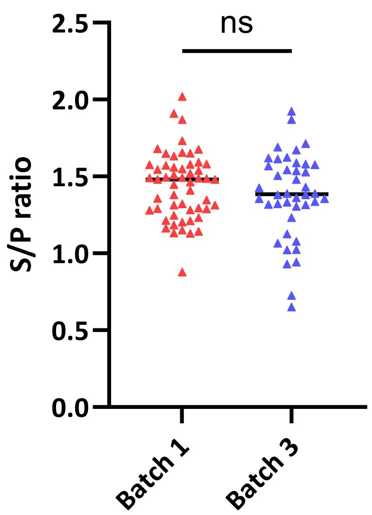

Supplement: Supplementary file 1 — Additional file 1. Pseudorabies-antibody levels of three-week-old piglets as determined by ELISA (S/P ratios). Distribution of S/P ratios for the offspring of the fourteen sows present in batches 1 and 3. Each triangle represents an individual. S/P ratios ≥ 0.4 are considered positive. ns = non-significant. [file 40813_2025_433_MOESM1_ESM.pdf]
