# Supplementary material for: A single recall vaccination lapse in sows triggers PRRSV resurgence and boosts viral genetic diversity
Source: Porcine Health Manag. 2025 May 8;11:26. doi: 10.1186/s40813-025-00433-w (PMC12063453; doi:10.1186/s40813-025-00433-w)

Additional File 2. Bayesian analysis of the ORF5 sequences obtained in this study. Posterior probabilities higher than 70% are shown. The colour-coded representation designates animals from Batches 1, 2, and 3 in red, green, and blue, respectively. Additionally, the identified clades from the complete genome analyses have been marked.

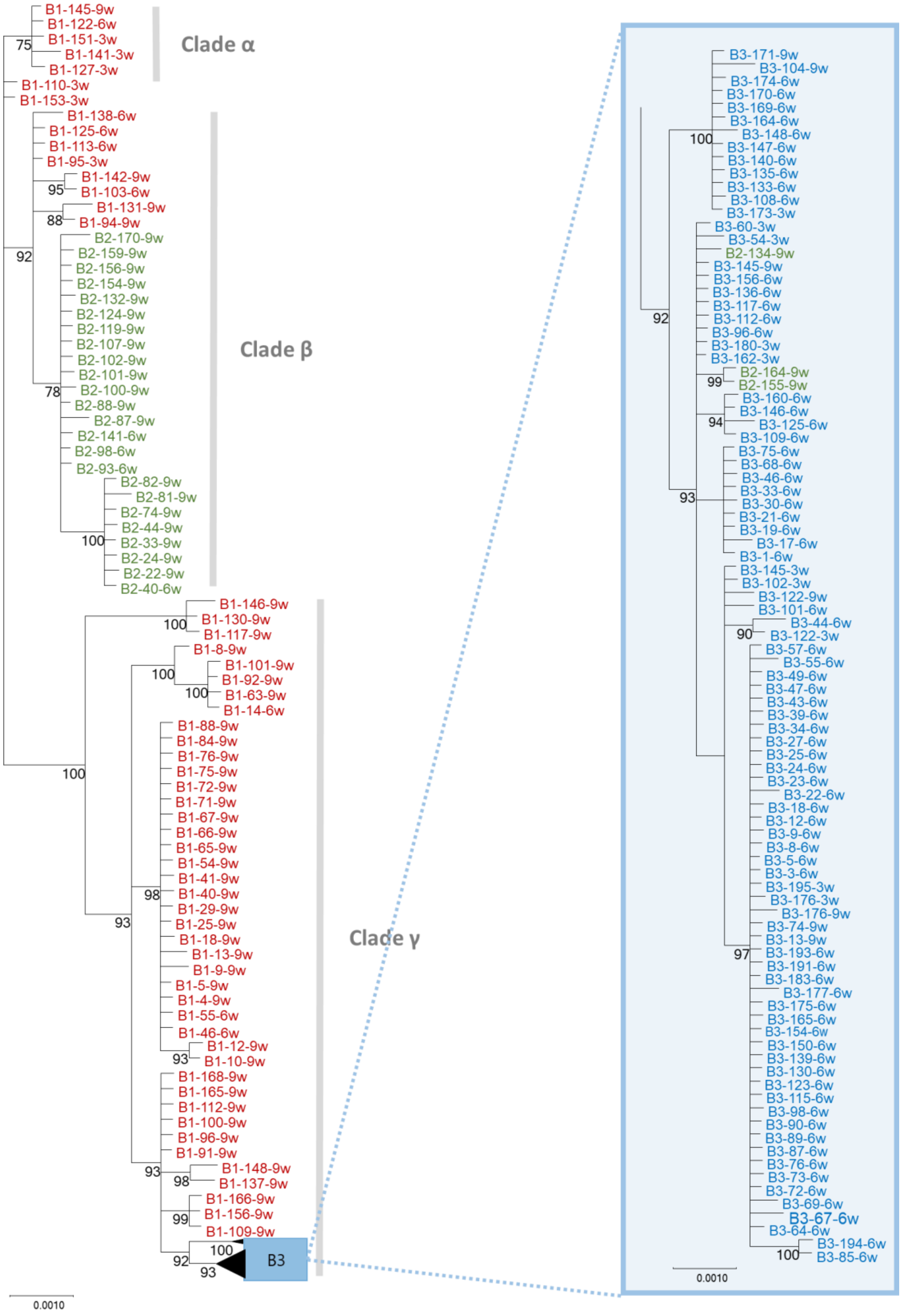

Supplement: Supplementary file 2 — Additional file 2. Bayesian analysis of the ORF5 sequences obtained in this study. Posterior probabilities higher than 70% are shown. The colour-coded representation designates animals from Batches 1, 2, and 3 in red, green, and blue, respectively. Additionally, the identified clades from the complete genome analyses have been marked. [file 40813_2025_433_MOESM2_ESM.pdf]
