# Supplementary material for: A single recall vaccination lapse in sows triggers PRRSV resurgence and boosts viral genetic diversity
Source: Porcine Health Manag. 2025 May 8;11:26. doi: 10.1186/s40813-025-00433-w (PMC12063453; doi:10.1186/s40813-025-00433-w)

Additional File 3. Recombination analysis of the whole genome sequences obtained in this study. Phylogenetic trees were constructed based on each recombinant fragment, incorporating the whole genome sequences of Batch 1 (in red), 2 (in green), and 3 (in blue). Individuals whose isolates from different ages clustered into a different clade are indicated with the same-coloured square.

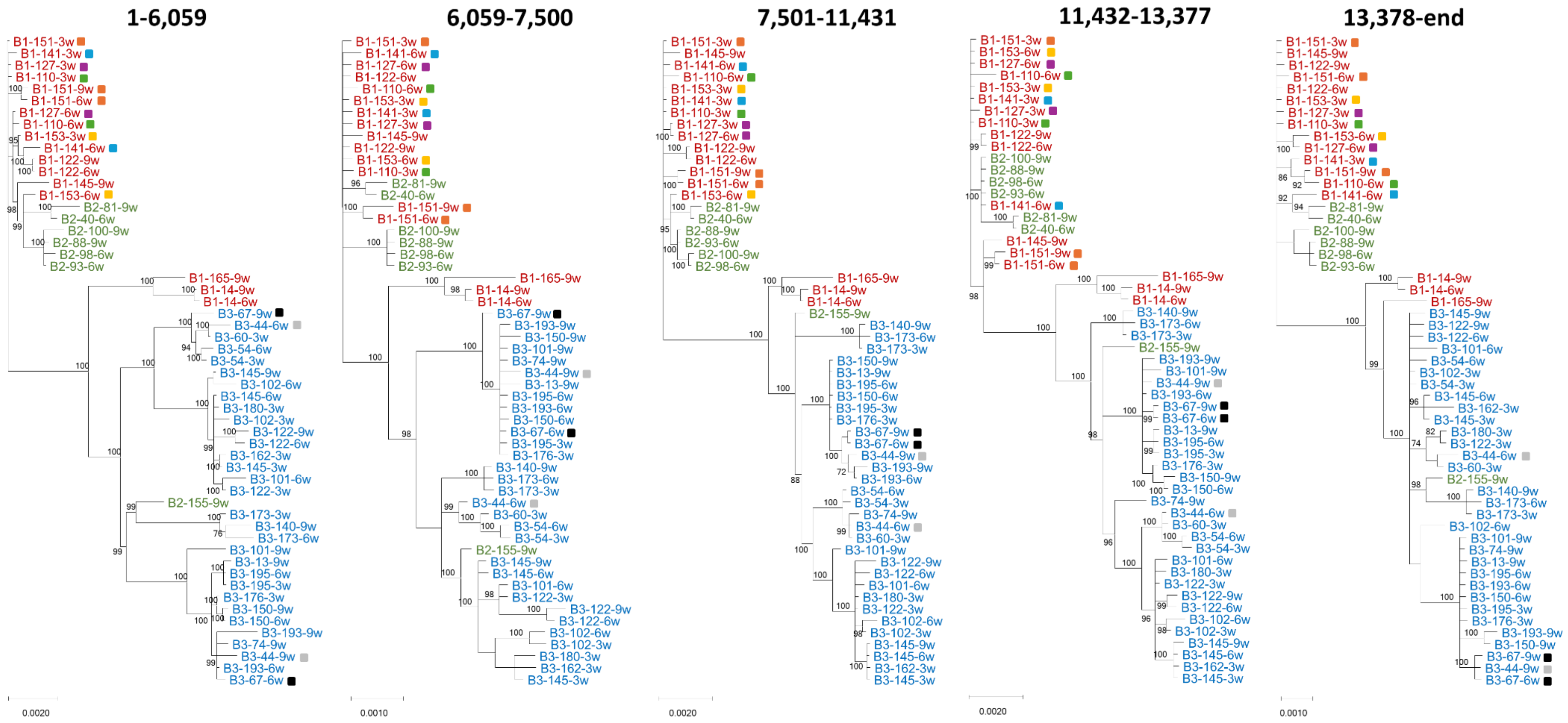

Supplement: Supplementary file 3 — Additional file 3. Recombination analysis of the whole genome sequences obtained in this study. Phylogenetic trees were constructed based on each recombinant fragment, incorporating the whole genome sequences of Batch 1 (in red), 2 (in green), and 3 (in blue). Individuals whose isolates from different ages clustered into a different clade are indicated with the same-coloured square. [file 40813_2025_433_MOESM3_ESM.pdf]
